# Feasibility and anticipated acceptability of community health worker-facilitated HPV self-sampling for cervical cancer screening around Lake County, Indiana

**DOI:** 10.1017/cts.2023.578

**Published:** 2023-06-23

**Authors:** Tiwaladeoluwa B. Adekunle, Alyssa Arreola, Sathveka Sembian, Raquel Castro, Layla Claure, Lara Balian, Natalia M. Rodriguez

**Affiliations:** 1 Brian Lamb School of Communication, Purdue University, West Lafayette, IN, USA; 2 College of Science, Purdue University, West Lafayette, IN, USA; 3 Weldon School of Biomedical Engineering, Purdue University, West Lafayette, IN, USA; 4 Department of Public Health, Purdue University, West Lafayette, IN, USA

**Keywords:** Health equity, HPV self-sampling, cervical cancer screening, community health workers

## Abstract

**Background/Objective:**

In light of calls to engage community health workers (CHWs) in the delivery of cervical cancer screening innovations, this study explores CHW perspectives on i) barriers to cervical cancer screening in a predominantly Hispanic community in Lake County, Indiana, the county with the highest cervical mortality in the state; and ii) the acceptability and feasibility of CHW-facilitated human papillomavirus (HPV) self-sampling as a means of reducing screening disparities.

**Methods:**

In 2021, in-depth interviews were conducted with 15 CHWs employed by Lake County community-based organizations including clinics, schools, and faith-based organizations.

**Results:**

Harnessing CHWs’ voices as insiders with knowledge of their communities’ health landscape, our analysis identified multilevel barriers to screening that spanned individual, interpersonal, and community levels of the socio-ecological model. CHW-facilitated HPV self-sampling shows promise of mitigating several barriers to cervical cancer screening. Privacy, time saved, and comfort were perceived to be facilitators for acceptability, with concerns about the novelty of this approach and trust in provider (as opposed to CHW) expertise emerging as key barriers. In terms of feasibility, synergies with existing CHW work, and some community members' prior experience with self-sampling were found to be facilitators, while CHW’s time limitations and self-efficacy in providing adequate medical support were areas of concern. Considerations for adoption included CHW training, gender concordance, safety, and respect, among others.

**Conclusion:**

This study provides critical insights from CHWs as key stakeholders on a screening model that directly engages them, which can inform implementation to increase screening in medically-underserved communities in the US.

## Introduction

Healthcare inequities are pervasive in the United States, with minoritized communities suffering from worse health access, lower quality care, and worse health outcomes than the majority population [1]. This trend also persists with cervical cancer as Hispanic women have an incidence rate that is 32% higher than that of non-Hispanic white women [2].

Indiana reflects nationwide disparities in cervical cancer, and Lake County, in particular, has one of the highest incidences (10 per 100,000 compared with statewide rate of 8.4/100,000) and mortality (4.0 per 100,000 compared with statewide rate of 2.7 per 100,000) rates for cervical cancer in the state [3]. Lake County is the second most populous county in Indiana with a population of over 499,000 people [4] and the highest proportion of Hispanic people of all Indiana counties (20%) [5]. Younger adults (25–44) and older adults (45–65) constitute 25% of the population each (for a total of 50%) [6]. Within Lake County, Hispanics have a disproportionately high cervical cancer incidence rate of 13.9/100,000, as compared to 11.1/100,000 for Black people and 7.8/100,000 for White people [7]. While there has been progress in reducing the prevalence of cervical cancer over the past few decades, ongoing screening disparities, healthcare disruptions related to COVID-19, and the potential for cervical cancer to be eliminated through screening and human papillomavirus (HPV) vaccination [8] necessitate innovative approaches to expanding screening access.

High-risk HPV (HrHPV) is the cause of most cervical cancer cases and can be prevented through vaccination and early screening [9]. Although the number of cases in the US has decreased in the past 40 years [10], the proportion of women without up-to-date screening increased from 14.4% in 2005 to 23.0% in 2019 due to socioeconomic and cultural factors [11].

There have been a variety of approaches to cervical cancer screening, with varying degrees of sensitivity, drawbacks, and advantages. Cytology-based screening (Pap smears) was historically the only method of screening, with clinicians taking and testing a sample retrieved from the cervix. However, because most cervical cancers are caused by hrHPV, HPV testing via self-sampling has been identified as an effective way to circumvent common barriers to Pap testing [12,13] and to screen women who are not currently being screened for cervical cancer [14]. Testing on self-collected samples can be as accurate as clinician-collected samples when used with hrHPV assays based on polymerase chain reaction [15]. HrHPV testing is also more effective than cytology-based screening and enables women to collect their own cervicovaginal samples even in nonclinical settings such as the home [15,16].

Efforts to increase screening in the US have been met by barriers, particularly among minoritized communities. A study among uninsured women eligible for free cervical cancer screening procedures found that cost (61.6% of respondents), fear of finding cancer (53.1%), and anxiety about the procedure (38.7%), were major barriers to screening [17]. Other barriers included embarrassment (25.6%), anticipation of pain (23.6%), and the presence of a male physician (19.7%). COVID-19 has also exacerbated screening disparities due to disruptions in preventative health care services and declines in cervical cancer screening [18]. In light of these disruptions, the President’s Cancer Panel has advocated for equity and resilience in cancer screening, recommending engaging community health workers (CHWs) and leveraging technological innovation in self-sampling as means of increasing screening uptake [19].

CHWs have become an increasingly prominent part of the healthcare landscape in the United States [20]. These professionals work as part of community-based efforts to provide health education and promote access to care, particularly in low-income and racially and ethnically minoritized communities [21,22]. There has been increasing attention to CHWs’ ability to increase cancer screening in their communities. Indiana, for example, lists engaging CHWs, patient navigators, and lay advisors as one of the strategies to increase the percentage of females screened for HPV by 2023 (Indiana Cervical Cancer Strategic Plan).

CHWs can be engaged in different ways during the screening process. They can help provide education [23], navigate individuals through the health system for Pap smear appointments, or help facilitate self-sampling, among other methods. One study found that a CHW educational intervention led to an increase in knowledge about cervical cancer, and self-efficacy for pap smear screening [23]. Other researchers conducted a study testing a multi-lingual intervention with CHWs who provided navigation services (tracking, scheduling appointments, etc) to the community members and found significant increases in cervical cancer screening uptake [24]. Likewise, another study conducted a randomized pragmatic clinical trial comparing three interventions from CHWs in the screening process: outreach, education, and navigation (for Pap smears) and education with the option to self-sample or Pap-smear [25]. This study found the CHW-driven option that included education with navigation to self-sample was the most effective, with 77% of women completing screening, as compared to 31% in the outreach group and 41% in the navigation (to Pap smear) group.

While self-sampling and delivery innovations (such as engaging CHWs) in HPV testing are believed to be an effective means of mitigating existing barriers, meaningful, deep consideration of community voices and structures are essential to the successful implementation of these processes [26]. As such, diverse community voices must be foregrounded, including CHWs and community members themselves. However, there is a dearth of literature that examines CHW’s perspectives on self-sampling, its fit into their scope of work, or their perceptions of how it might be received by their communities. CHWs could provide insight into feasibility, and the extent to which CHW-assisted self-sampling could successfully be implemented within the community, as they have a close understanding of their community’s healthcare systems, educational services, and barriers to healthcare access [27]. With this knowledge, CHWs could help determine the resources their community would need to adequately support the implementation of a self-sampling intervention. Also, given their typical in-group identity and familiarity with the socio-cultural contexts of their community, CHWs can provide insight into acceptability, and their community’s willingness to try new screening approaches.

This study conducts a qualitative exploration of CHWs’ perceptions of CHW-facilitated HPV self-sampling for cervical cancer screening in Lake County, Indiana. Specifically, this paper explores CHW perspectives on barriers to cervical cancer screening, the feasibility and acceptability of CHW-led HPV self-sampling strategies, and key considerations for implementation of this screening modality. The findings of this study are relevant for increasing screening uptake in Lake County and similar contexts across the United States.

## Methods

### Procedure

Semi-structured in-depth interviews were conducted with CHWs to glean insights into barriers to cervical cancer screening in underserved Indiana communities. CHWs were recruited passively through community partners’ mailing lists. An IRB-approved email (IRB-2021-1385) was sent out inviting potential participants to participate in a 30–60-minute interview. CHWs who expressed interest in participating were contacted. Participants received a $25 electronic gift card as compensation at the completion of the interview. Recruitment of CHWs began in Lake County and then expanded to the South Chicago area after the study team learned through initial interviews that some community members seek care from Chicago. We conducted virtual interviews (using Zoom) with a sample of 15 CHWs from October to November 2021. Interviews were audio recorded and transcribed using a digital platform, Otter.ai. Transcripts were reviewed and edited for accuracy by research assistants.

### Interview guide

The questionnaire was formulated and refined by the study team. The final interview guide included 11 questions supplemented with probes for in-depth exploration of emerging topics. The guide included questions about CHW’s typical workday, health concerns and barriers in their communities, and their perspectives on benefits and pitfalls of CHW-facilitated self-sampling. The questions were designed to gain insight into: i) multilevel barriers to care guided by the socio-ecological model (SEM), and ii) the range of factors influencing feasibility and acceptability of self-sampling in these communities. The complete interview guide is available in Appendix A.

### Qualitative coding and analysis

Deductive and inductive coding was utilized in the analysis of our data [28]. Members of our research team developed a codebook based on our research objectives and interview guide. Trained research assistants used the qualitative data analysis software NVivo for the initial coding of the qualitative transcripts. Coders discussed their independent coding to discuss any differences in coding and reach consensus. The coded transcripts were then analyzed by three trained research assistants to collectively identify themes. SEM was used as a guiding framework to analyze barriers and facilitators to healthcare and cervical cancer screening across individual, interpersonal, and community levels [29].

## Results

**Figure 1.**
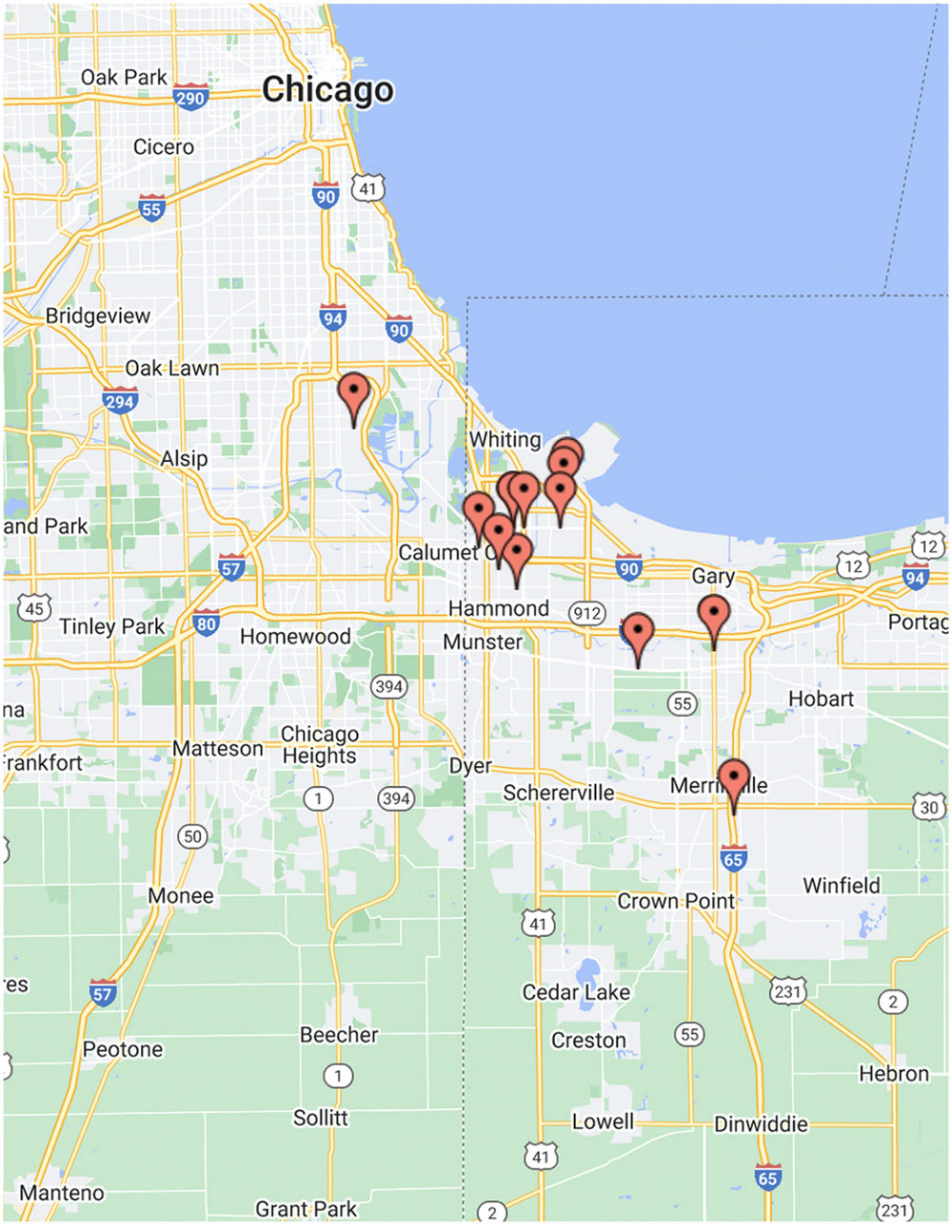
Distribution of study participants’ areas of employment.

Between October and November 2021, 15 CHWs were recruited to participate in 30-to-60-minute virtual interviews. As shown in Table 1, of these 15 participants, 14 (93%) were female, 11 (73%) were Hispanic, 2 (13%) were White, non-Hispanic, and 2 (13%) were Black, non-Hispanic. The majority of the CHWs interviewed for this study are Hispanic. This reflects the populace of Lake County, which has the second-highest proportion of Hispanics in the state of Indiana [5]. CHWs were employed across a range of organizations, with a little over half (53%) engaging in clinic-based work. For their most recent title, 40% reported “community health worker,” while another 40% reported a combination of outreach, community case managers, or engagement coordinators. 53% of participants reported having 1 to 5 years of prior experience in their field. As shown in Figure 1, the study participants’ areas of employment were spread across the northern Indiana and the Chicagoland areas, with a majority (60%) based in Lake County, Indiana.

**Table 1.** Participant demographics

Demographic information	N	%
Gender		
Female	14	93
Male	1	7
Current/ most recent job title		
Community health worker	6	40
Navigators	2	13
Community relations	1	7
Outreach/Community Case Manager/ Engagement coordinator	6	40
Years of experience		
1–5 years	8	53
6–10 years	4	27
11–15 years	1	7
16–20 years	2	13
State		
Indiana	12	80
Illinois	3	20
County		
Lake County	9	60
St. Joseph	2	13
Cook	3	20
LaPorte	1	7
Race/ethnicity		
White, non-Hispanic	2	13
Hispanic/Latinx	11	73
Black, non-Hispanic	2	13
Place of work		
Clinics	8	53
Health centers/organizations/programs	5	33
School	1	7
Church	1	7

### CHWs’ role in mitigating healthcare and cervical cancer screening barriers in Lake County

CHWs discussed multilevel barriers to healthcare and cervical cancer screening in their communities, and their work to mitigate these barriers, summarized in Table 2.

**Table 2.** CHW’s role in mitigating multilevel barriers to healthcare

SEM levels	Barriers	CHW efforts and supporting quotes
Individual	Financial barriers	Provide help to patients without insurance “*In my job position I assist patients that come to the clinic and don't have health insurance… I try to help them get health insurances and also refer them to other services they need within the community*.”
	Difficulty navigating healthcare system in Indiana	Support patient’s navigation in the health care system “*I have been working helping with registration. I help them navigate through the online by saying where to click, go to the next page, click here.”*
	Immigration/Documentation status	Supporting undocumented individuals *“I work with people who are undocumented, and we try to support and fight for people to obtain license and people who don't have documented.”*
	Language barriers	Interpretation *“ As a translator in the clinic I can aide in …giving them information, that scheduling, helping schedule appointments, that something I can do.”*
	Lack of knowledge about benefits of screening	Providing health education “*I encouraged them about the pap smear, how its helped people and helps save people’s lives…the numbers are out there where cervical cancer detected in time have saved many women, and you know pap smear, can save you from other things, other health conditions as well.”*
Interpersonal	Mistrust/Distrust	Working to Foster Trust “*I knew that behind the phone call there’s more issues that I needed to address of what’s keeping her from making appointments. And those conversations without them knowing they opened up to me*”
Community	Housing insecurity	Finding adequate housing *“Resources to the housing area so that you can make sure that you’re able to apply for housing assistant or look for somewhere to rent or whatever the case is.”*
	Transportation	Providing transportation services *“Like, people don't have transportation to get back and forth to the doctor or an appointment, things like that, I can take them bus cards…I pick up homeless families from like living in parks and taking them to shelters.”*
	Scarcity of healthcare organizations	Outside of scope of work
	Lack of resources to raise awareness	Outside of scope of work

CHW, community health worker; SEM, socio-ecological model.

### Individual level barriers

At the individual level, CHWs reported that financial barriers impede community members from being able to access care, “*A lot of people tend not to go to the hospital or a doctor, because they can't pay out of their pocket or…they don't have the resources necessary*….”

Another barrier to healthcare and cervical cancer screening includes the perceived difficulty of navigating healthcare in Indiana, leading some to opt for care in nearby Chicago. As one CHW described: “*Easier to get healthcare access in Chicago than it is in Indiana. One of the reasons is that they used to reside in Illinois, or they know somebody. But in Indiana, it just seems to them that it is not user friendly, or they can not access [to] healthcare…*”

Compounding these barriers is the limited knowledge community members hold about their susceptibility to diseases, the benefits of screening, and the resources that might be available in the community to support them: “*A lot of the community members are not aware of all the resources that are there to help them, financially, medically, or mentally*” According to CHWs, most women only had screenings when they were pregnant or had a baby: “*If they don’t have a baby or pregnancy they will not be seen for paps because “why do I need to go see you”*?.” Some community members were also unaware of the benefits of screening and had a fear of receiving bad news, “*There is a lot of hesitation of “I don't want [to] go because then I’m just going [to] get some bad news”*.”

Lack of knowledge about resources available was heightened for undocumented individuals who may feel discomfort with seeking care: “…*I have to ask them sometimes …so that they know that even though they might not qualify for some things, there still some resources that they might be able to get…*.”

Finally, language was reported as a barrier that exacerbates difficulties with education. Due to information about screening and cervical cancer not being written in multiple languages, some community members do not learn about it, “*We try and provide all our filers in both English and Spanish, but …not every place does that*.”

### Interpersonal level barriers

CHWs reported that lack of trust has impacted the patient and healthcare provider/services’ relationship: “*I just feel there is medical mistrust*.” Another perspective underlines the challenge of reaching patients who have a distrust: “*its is a challenge at times for clients to trust you and to accept the help. To even answer the phone when I call them.*”

### Community level barriers

At the community level, a scarcity of healthcare organizations, clinic closures due to lack of resources, and challenges with transportation as barriers to healthcare and cervical cancer screening.

One CHW drew attention to the scarcity of healthcare organizations: “*We do not have many clinics in this area, it is a healthcare desert in a way…the population we serve, they rely on us, the community clinics.”* Another CHW mentioned that some clinics had shut down: “*Main reason that prevents members from accessing the health care they need is hospital and clinic closures*.”

Likewise, transportation was reported by CHWs to be a major impediment to community members gaining access to care: “*This whole area has a big disproportionate thing with the bus systems,… There need to be more buses…*”

Furthermore, housing insecurity impacted healthcare access in different ways, one of them being that it made outreach more difficult. As one CHW described, “*Very complicated to go to the abandoned buildings because that is their home, they have to invite the person who wants to do the outreach to see where they live. But we have a community garden next to our office so the majority meet there.”*

Finally, CHWs reported a lack of resources to raise awareness among community members: “*No kinds of literature out there to make them aware that this is something that they should be checking as a woman*.”

### Role of CHWs in addressing health barriers in the community

CHWs described the roles they play to address health and cervical cancer barriers in the community, primarily during home visits and clinic-based work. Table 2 maps CHW efforts to a number of barriers, with corresponding illustrative quotes. While CHWs work to address many barriers at the individual and interpersonal levels, many barriers at the community/structural levels are beyond CHW’s capacity to address, necessitating coordinated efforts by stakeholders across state government and the healthcare system.

The majority of these multilevel health barriers impede cervical cancer screening in the community, and as such, CHWs play various key roles in mitigating screening barriers through their engagement with community members. Below, we discuss our findings on CHW’s perspectives of the feasibility and acceptability of a self-sampling innovation to facilitate cervical cancer screening in their communities.

### CHWs’ perspectives on feasibility and acceptability of CHW-facilitated HPV self-sampling

CHWs were presented with a hypothetical scenario in which CHWs facilitate the delivery of HPV self-sampling. The CHWs then provided their insight into key barriers and facilitators to the feasibility and acceptability of implementing this model in their communities, summarized in Table 3.

**Table 3.** CHWs’ perspective and perception view of community members on acceptability and feasibility of self-sampling

	Acceptability	Feasibility
Barriers to CHW-Delivered Self-sampling	*CHW’s perspective* Lack of self-efficacy Issues gaining trust of community members	*CHW’s perspective* Lack of time in work day
	*CHW’s perception of community members:* Concerns for novelty of the intervention Trust in provider’s expertise	*CHW’s perception of community members:* Issues with privacy in the home (Lack of physical space) Issues with self-sampling execution and community members following directions
Facilitators to CHW-delivered self-sampling	*CHW’s perception of community members’ acceptability:* Save time Increase comfort Reduce barriers related to transportation Privacy offered by personal space	*CHW’s perspective:* Educational component offered by CHWs’ guidance Leverage existing relationships with community members Synergies with model of existing work

CHW, community health worker.

### Acceptability of CHW-delivered self-sampling

#### Facilitators

CHWs perceive that facilitating the delivery of self-sampling at home presents benefits relevant to its acceptability to key stakeholders (community members who will be using the self-sampling device, and CHWs who will be providing assistance in this process). From the perspective of CHWs who will be providing assistance, these benefits include the opportunity to provide education and guidance to community members during the process, the chance to leverage existing relationships with community members, and the synergies of this model with existing work. CHWs also anticipated that for community members who will be using the self-sampling device, self-sampling would be more comfortable, save time, and reduce barriers to access.

Self-sampling (if done at home) increases access to sampling because CHWs can reach clients directly, avoiding challenges related to transportation and other factors mentioned in Table 1. CHWs highlighted the importance of meeting individuals directly as “*sometimes people are not out there in the fairs or …they do not have the means to transportation to get to clinics …*” In addition to the low barriers to access, CHWs described how sampling at home presents an additional benefit by offering some individuals privacy “*…no one will be here from 10 to 12. You can come over then. I have some privacy”*.

Another CHW described the home setting as being the location *“where [community members] are most comfortable*,” making them more “welcoming” to CHWs. CHWs also shared the belief that self-sampling eases the inherent discomfort of the pap test experience and presents a more appealing and less invasive alternative, “*…some people are just more comfortable being able to do that themselves and not having to get undressed for somebody to get checked. If it’s not necessary.*” Another perceived benefit is the ability for CHWs to provide guidance during the self-sampling process, increasing the likelihood that the process would be carried out correctly, “*…it would be good in that it would definitely get people to do…[the screening] and I think it ensures the best chances of doing it properly.*”

#### Concerns

While CHWs reported a few factors that contribute to the acceptability of the model of self-sampling discussed here, they also expressed concerns that impede acceptability. These are the complexities of privacy for some community members, trust in the providers’ expertise, and the novelty of the test. These themes are also seen in Table 2.

Firstly, CHWs reported that although some community members may have more privacy at home, for others, being at home does not offer the same level of privacy: “*there’s women that, “why am I going to talk about my breast when there’s my kid over there somewhere?” Or there’s three or four families living in the household…”* One CHW elaborated on this point, drawing parallels with past telehealth visits with providers: “*…When we were doing video visit checks with the provider, the women didn't feel comfortable showing breast or talking about breast when their kids were at home….”*

Secondly, CHWs emphasized that individuals may feel less confident in the test because they trust and prefer the expertise of providers: *“Spanish population…believe in the providers, I think that they do not want to do self-sampling, I think that they rather have their provider, to tell them they’re fine, or this is something that we need to fix or cure….”*

Thirdly, CHWs may not be able to gain the trust necessary to be heard and understood by individuals. When asked about the anticipated challenges of CHW-facilitated self-sampling, one CHW responded: *“Being trusted enough, being trusted enough to be heard.”*

### Feasibility of CHW-facilitated self-sampling

CHWs shared the factors relevant to the feasibility of CHW-facilitated self-sampling, both from the vantage point of CHWs as well as from that of community members.

#### Facilitators

CHWs considered providing education to be in line with their current work at the clinic: *“ I feel like that’s what we do in the clinic with other education but it’s actually in the person’s home”*. Another CHW expressed that they are willing to be trained on new screening technology: *“Something that I can learn to add another feather to my hat and as it helps the community that I’m servicing, and that my whole goal is to help those in need.”*

CHW’s perception of community members underlined the belief that women who already have experience with self-sampling would be more receptive: *“I think that they do self-sampling here… I know swab, like, not necessarily a Pap smear, but something similar to that way they can self-swab…They do that now the clinic.”*

#### Barriers to feasibility

From the CHW’s perspective, self-efficacy and lack of time, impede the feasibility of self-sampling.

CHWs are not trained to provide healthcare and expressed discomfort at performing what they view to be medical procedures (in people’s homes): *“I wouldn't even feel comfortable giving extra medical advice, or I mean somewhere where I think I should pull in a nurse or a doctor or nurse practitioner…”*

In addition, CHWs may also not have the time to go to individual homes, particularly if it is not already a part of their routine to do so*: “…if you're sending the CHW out to every house but they have all these other things they're doing, they may have issues. But if they're already in the house I do not really see a negative to it.”*

For community members, CHWs expressed that improperly followed guidance restricted feasibility. One CHW noted that even during the home visit, individuals may still not follow their guidance: *“…even though you provided it doesn’t mean the person’s going to do it. So even if you give it to them, they’re at the house, they may still not do it.”*

### Considerations for implementation of CHW-delivered self-sampling

CHWs recommended six areas of consideration for the successful implementation of CHW-delivered self-sampling to benefit communities.

- *Provide training for CHWs*

CHWs mentioned that they would need to have the pertinent knowledge in order to support people through testing: *“The CHW would have to be knowledgeable in case that it is positive in order to support and be prepared to refer to the needed resources.”* If community members were to complete self-sampling on their own, CHWs also point out the need for CHWs to be well-trained: *“I believe they would if they were properly trained them how to do it… If they give me the education then I would feel more comfortable doing it myself.”*

- *Raise awareness, connect people to resources*

CHWs described that it would be important to educate people about HPV and cervical cancer, connect them to appropriate resources and ensure that they feel comfortable and consent to CHW assistance. As one CHW described, *“I think it’s a condition that once you're aware of it.., you know you'll take it seriously. And I think it’s the way you approach and inform them of this, this can make them feel comfortable and make them understand what resources are available to them..”* Thus, CHWs recommended creating supplementary programing and outreach efforts geared toward education: “*we can host like women’s events and … give education and you know give maybe like a lunch and learn, implement different ideas to motivate people to attend and do like a series of them so the women that have attend have an opportunity to invite their friends and loved ones to this,…”*

Another CHW emphasized the importance of offering resources after self-sampling, particularly to clients who are undocumented: *“cannot just say here’s a test and you’re positive and that’s it. No, you need to give more, connecting them with having the right resources. in their language, then I think you'd be able to help people get the care they need, preventive care and treatment.”*

- *Show respect*

Informed consent and respect are also highlighted as important for consideration by the CHWs. Women may be receptive to CHW delivery if CHWs gain permission first: *“ The reality is if you ask permission from the person and you explain why you want to see them, I don’t think there would be any problem in speaking with them because they already accepted to receive the information. The problem would be to go to someone’s house without announcing yourself.”* Another CHW supported this view: *“Communicating with them efficiently and respectfully, we can help get this done”*

- *Consider gender concordance of CHWs with community members*

CHWs also mentioned how gender concordance may impact individuals’ comfort during self-sampling: *“I think what would work is that it would be women taking care of women and men taking care of men because a woman grandma would be embarrassed to speak about her sexuality and practices. I think there is caution and I think they should go directly to a woman and a man to a man.”* Thus, implementation of self-sampling should prioritize gender concordance of CHWs with community members.

- *Ensure safety of CHWs*

From an organizational perspective, at-home self-sampling facilitated by CHWs presents general as well as COVID-19-specific health and safety considerations for both clients and CHWs. As one CHW stated: *“… making sure that the health and safety of the workers is considered and how best to do that too, they go out in teams to gather what are the red flags that maybe that might not be a safe home to visit for some reason, thinking about both environmental and like physical.”* Another CHW expressed a similar concern: *“And there are some that there could be some potential dangers as well, you know, I'm talking about like physical danger like a, like a dog or something might not be able to, might not be tamed, or you know, put in another room or something like that.”*

- *Consider context*

The context of delivery is highly consequential for individuals’ comfort. While some individuals have more privacy at home, this study found that other individuals actually have less privacy at home, and feel uncomfortable discussing reproductive health with their family members in close proximity. From the CHW’s perspective, however, being at the clinic, with access to the expertise of providers may foster confidence in their ability to successfully facilitate the self-sampling process. Thus, public health practitioners and community health organizations should be responsive to the particular contextual needs of both the individuals taking the test and the CHWs providing assistance.

## Discussion

In light of national calls to engage CHWs in screening delivery and leverage self-sampling to increase screening, the current study qualitatively explores CHW’s perspectives of (a) barriers to healthcare and cervical cancer screening in their communities and (b) the feasibility and acceptability of CHW-facilitated HPV self-sampling. Harnessing CHWs’ voices as insiders with knowledge of their communities’ health landscape, our analysis identified multilevel barriers to screening that spanned individual, interpersonal and community levels of the Socio-Ecological Model. While various studies explore the cervical cancer screening and/or HPV self-sampling perspectives of people with cervixes [17,30] and others recognize the effectiveness of engaging CHW’s in the self-sampling process [25,31], few studies foreground the insights of this critical stakeholder group.

At the individual level, CHWs reported financial insecurities, difficulty navigating healthcare, immigration status concerns, language barriers, and a lack of knowledge about the benefit of early screening (and fear of finding something wrong) as barriers to healthcare and cervical cancer screening. At the interpersonal level, CHWs identified lack of trust as the main barrier to healthcare. At the community level, clinic closures, housing insecurity, inadequate transportation, and scarcity of healthcare organizations were revealed as key barriers to healthcare. These challenges made CHW outreach more difficult and decreased the likelihood that individuals would receive much-needed healthcare and screening. These challenges highlighted by CHWs align with previous findings on multilevel screening barriers in underresourced communities [17,32,33].

Our analysis also reveals the role of CHW’s in mitigating these multilevel barriers to health and screening. This supports findings from previous studies that emphasize CHWs’ effectiveness in supporting community members in navigating healthcare barriers [34,35]. However, we also highlight that while CHWs can more easily address individual-level barriers (e.g., language) barriers at the community level, (e.g., clinics closing due to lack of resources) are beyond CHWs’ capacity to solve. This finding underscores the need for interventions that address both individual-level barriers as well as broader structural issues that are detrimental to cervical cancer screening.

In assessing the perceptions of feasibility and acceptability of CHW-facilitated self-sampling, this study found that this model of delivery is a promising way of increasing screening uptake in underserved communities. Previous studies have emphasized the utility of engaging CHWs and using self-sampling to increase screening, however, few have explored the potential of both of these methods in tandem, from the CHW’s viewpoint. In seeking to bridge this gap in the literature, we have found that synergies with CHWs current work, CHWs current rapport, and building trust with community members all serve to enhance the feasibility of this mode of delivery. Likewise, CHW’s perspectives on the time saved, privacy (in some cases), added convenience, and circumvention of the need for transportation enhance the acceptability of this approach. These benefits for acceptability underscore findings in literature on how self-sampling serves to mitigate barriers to screening and increase screening uptake (Murphy *et al.*, 2016). However, this study adds an important layer of nuance in underscoring that some established benefits of self-sampling, such as privacy do not apply to community members who may, for example, have family members at home and feel uncomfortable discussing reproductive health in their presence. Similarly, while self-sampling eliminates the necessity of what previous studies have found to be uncomfortable doctor’s visits for pap smears [12,18], our study highlights that some community members appreciate and trust the expertise of a healthcare provider and may not feel confident in undertaking the process.

Finally, this study reports CHW-informed considerations for CHW-facilitated HPV self-sampling. These considerations include: providing training for CHWs, raising awareness and connecting people to resources, showing respect, and considering gender concordance. The importance of gender concordance specifically has been highlighted in previous studies [36,37]. Thus, it is important for public health practitioners and community health organizations to note the importance of gender in the delivery of CHW-facilitated self-sampling.

In foregrounding the voices of CHWs, a limitation of this study is that it does not directly incorporate the perspectives of community members themselves on CHW-facilitated self-sampling. While CHWs have important insight into the experiences of people within the community and can detail their perceptions of community members' attitudes toward self-sampling, they cannot fully anticipate how community members would respond to their help. Future studies will focus on understanding community members’ perspectives. Another limitation is that CHWs in this study responded to a hypothetical scenario in which they would facilitate self-sampling. While CHWs are experienced in similar types of work and can anticipate the complexities of this hypothetical process, carrying out the process, in reality, may still present other practical challenges and opportunities not detailed here. This is another consideration for future work.

Furthermore, this study focuses on self-testing for HPV with CHW facilitation. HPV self-testing presents relative advantages and drawbacks that are important to note. While self-testing is more sensitive than cytology-based screening and can expand screening access, cytology-based screening is preferable for younger women who are more likely to have nonthreatening HPV infections. Another important consideration is therefore that community members have adequate follow-up following a positive test, in order to alleviate anxiety and or provide guidance on next steps.

## Conclusion

From the perspectives of CHWs, we found that a CHW-led approach to HPV self-sampling is considered both a highly feasible and acceptable cervical cancer screening innovation. Although CHWs identified challenges to implementation, with the consideration of six areas of focus highlighted in this study, this new screening approach shows promise of improving screening rates and cervical cancer incidence within medically underserved and racially/ethnically minoritized populations in Lake County, Indiana, and similar contexts across the US.

## Data Availability

To protect the privacy of participants, the data cannot be made available.

## Supplementary material

For supplementary material accompanying this paper visit https://doi.org/10.1017/cts.2023.578. click here to view supplementary material
