# Supplementary material for: Feasibility and anticipated acceptability of community health worker-facilitated HPV self-sampling for cervical cancer screening around Lake County, Indiana
Source: J Clin Transl Sci. 2023 Jun 23;7(1):e157. doi: 10.1017/cts.2023.578 (PMC10388433; doi:10.1017/cts.2023.578)
Supplement: Supplementary file 1 [file S2059866123005782sup001.docx]

**Appendix A**

**Community Health Worker Interview Guide**

1. Tell me about you and your org.

2. How long you have been a CHW?

a. Why and how did you become a CHW?

b. What trainings have you received as a community health worker?

3. Describe your typical workday as a CHW.

a. Describe your range of responsibilities.

b. Clinic-based? Home visits? Outreach?

4. Describe the community you serve.

a. Are you from this community?

b. Common health concerns of the community?

c. Biggest challenges in community?

d. Where do people in your community spend their time? Orgs/services they use?

5. Do you feel like your community has appropriate access to healthcare?

a. In your opinion, what are some of the main reasons that prevent community members from accessing the healthcare they need?

b. Where do your community members go when they are sick?

c. How do they pay?

6. What is the best means of communication for conducting outreach and informing community members?

a. When first approaching community members, are there specific methods that work best for engaging them? Describe them.

b. How do you follow up with community members who need or have received care?

c. Have you been part of any health education activities?

d. Challenges to this?

7. In your opinion, how receptive are the women in your community about speaking and learning about sexual and reproductive health?

8. What do you know about cervical cancer?

a. Have you had any experience (personal, family/friends, within the communities you work in) with cervical cancer in the past? And if so, could you describe it?

b. Do you know of ways to prevent cervical cancer?

c. What methods are you aware of for detection of cervical cancer? What do you know about ways to treat the disease?

d. What resources are available to the community for cervical cancer screening… diagnosis and treatment?

e. Do you think the women in your community know about cervical cancer and ways to prevent/detect? Know about resources?

f. Barriers to screening?

i. Cultural barriers? Beliefs? Education?

g. Facilitators to screening?

h. Have you ever encouraged the women in the community you work in to seek a pap smear or pelvic exam?

i. What would you like to learn about this disease? Do you have any questions about cervical cancer?

9. Do you or have you ever used any technology to help you in your community health work?

a. Probes: iPads, tablets, blood glucometers, pregnancy tests… other diagnostic tests?

10. Would you be willing to be trained to use a new technology that could help women understand their risk for cervical cancer?

11. [Description of the hypothetical intervention- CHW-delivered RDT for HPV screening with self-sampling] Do you think this is a good idea? Why or why not?

a. Have you ever heard of self-sampling? If so, what do you know about it?

b. Benefits to this?

c. Challenges/pitfalls to this?

d. Would women be receptive to self-sampling?

e. Cervical cancer education?

f. At-home testing?

g. Would clinic-based rapid testing be useful in your opinion?
